# Satisfaction with Health Facility Personnel Among Older People with Disabilities in Chile: An Observational Study Based on the 2024 DISCA Survey

**DOI:** 10.3390/ijerph22071103

**Published:** 2025-07-13

**Authors:** Elena S. Rotarou, Dikaios Sakellariou, Rafael Pizarro-Mena

**Affiliations:** 1 National Department of Public Health, Faculty of Medicine, Universidad San Sebastián, Los Leones Campus, Santiago 7500000, Chile; 2 Millennium Nucleus Studies on Disability and Citizenship—DISCA (NCS2022_039), Universidad San Sebastián, Los Leones Campus, Santiago 7500000, Chile; 3 Interuniversity Network on Healthy Ageing in Latin America and the Caribbean (RIES-LAC), Universidad San Sebastián, Los Leones Campus, Santiago 7500000, Chile; 4 Millennium Nucleus Studies on Disability and Citizenship—DISCA (NCS2022_039), European University Cyprus, Nicosia P.O. Box 22006, Cyprus; 5 Department of Health Sciences, European University Cyprus, Nicosia P.O. Box 22006, Cyprus; 6 School of Healthcare Sciences, Cardiff University, Cardiff CF14 4XN, UK; 7 Faculty of Rehabilitation Sciences and Life Quality, Universidad San Sebastián, Los Leones Campus, Santiago 7500000, Chile

**Keywords:** older people, disability, patient satisfaction, access to healthcare, Chile, health facility personnel

## Abstract

Achieving health equity for people with disabilities requires addressing the barriers that they face when accessing healthcare. Older adults with disabilities may experience compounded disparities, yet little research has explored their satisfaction with health facility personnel, including non-healthcare staff. This study examines differences in satisfaction with health facility personnel between younger (18–59 years) and older (60+) adults with disabilities in Chile. Data from the 2024 Disability and Citizenship (DISCA) survey were analysed using chi-square tests to examine differences between younger and older people with disabilities with regard to their satisfaction with health facility personnel. Ordered logistic regressions were employed to predict high satisfaction, given socioeconomic and health-related variables. Findings indicated that a higher percentage of older adults with disabilities reported high satisfaction with health facility personnel compared to younger adults. Ordered logistic regressions confirmed that older adults had greater odds of reporting high satisfaction with doctors (OR: 3.83), other health professionals (OR: 4.66), paramedical technicians (OR: 4.31), and administrative staff (OR: 3.13). These results suggest that age influences satisfaction levels among people with disabilities, potentially due to varying expectations, experiences, or interactions with health facility personnel. Understanding the underlying reasons for these age-related differences is essential to inform policies and practices that ensure equitable, person-centred care for people with disabilities across the life course.

## 1. Introduction

Achieving global and national health goals requires health systems to prioritise equity in healthcare, particularly for disadvantaged groups, such as people with disabilities. As populations age, the intersection of disability and older age further compounds health disparities, increasing the risk of inadequate access to care and poorer health outcomes [1]. With 1.3 billion people—16% of the global population—living with disabilities [1], health systems must explicitly address their needs to ensure inclusive and equitable healthcare. Coordinated efforts are essential to understanding the specific health challenges faced by this population and developing targeted strategies to effectively meet their needs.

Following global trends [2], societies across the Latin America and the Caribbean region are ageing, with the proportion of people aged 60 and over projected to be between 25% and 30% by 2050 [3]. While in Chile, the percentage of people over 60 years of age reached 18% of the total population in 2022, this percentage is expected to reach 23% by 2030 and 37% by 2060, making it the country—together with Uruguay—with the oldest population in the region [4]. The evidence also shows a strong association between age and disability, with the global prevalence of disability rising from 5.8% in people aged 0–14 years to 34.4% among people aged > 60 years, while it is even higher for people over the age of 80 [1]. This fact, coupled with the demographic transition towards ageing societies, especially among high-income countries, calls for a close look at the needs of this demographic group.

The World Health Organization (WHO) recognises that meeting the health needs of the population requires a focus beyond service coverage and affordability to also include the quality of services and how well they respond to people’s needs [1]. The availability and affordability of services need to be complemented by service user satisfaction to ensure utilisation. Satisfaction refers to the degree to which service users think a health system meets their perceived needs, and it encompasses several dimensions, including perceived efficiency, timeliness, and the provision of care in a respectful manner, taking into account the wishes and needs of service users [5,6]. Satisfaction is related to the expectations people have of a health system: high satisfaction might be linked to low expectations rather than positive experiences and not necessarily to good quality of services. While there is no agreement on the components of patient satisfaction, the importance of interactions between service users and health facility personnel is consistently found to be an important determinant of satisfaction with services, as it is through these interactions that service users experience the health system [7].

Poor service user satisfaction can lead to low utilisation, including non-engagement with services and low adherence to healthcare advice, resulting in worse health outcomes and higher burden to health systems [8,9,10,11,12,13]. Evidence demonstrates that people with disabilities—often compounded by generally lower coverage of health insurance, lower socioeconomic level, and higher healthcare needs—are less likely to be screened for cancer [14,15], often due to intangible barriers related to healthcare workers’ attitudes [16]. Women with disabilities are less likely to receive appropriate information regarding mammography and Pap tests, while previous negative experiences might lead disabled women to skip screening procedures altogether [17].

Chile—a signatory to the Convention on the Rights of Persons with Disabilities—CRPD (2007)—has a dual health system in which health insurance is divided into public (Fondo Nacional de Salud—FONASA) and private (Instituciones de Salud Previsional—ISAPREs). These two providers are not complementary, and all people have to choose their affiliation with one or the other (there is a separate health system and insurance for people affiliated with the Armed Forces and Security, which comprises approximately 3% of the population). This two-tier health system has created a stratification of access to health: people with a higher socioeconomic status are affiliated with one of the ISAPREs (about 15% of the population), while people with a lower socioeconomic status or belonging to a disadvantaged group—such as people with disabilities, older people, people with chronic conditions—are affiliated with FONASA (about 80%). 

However, the COVID-19 pandemic, coupled with significant increases in ISAPRE premiums, has led to a notable shift in Chile’s health insurance landscape. In 2023, 81% of the population was enrolled in FONASA, the public health insurance system; this includes 84% of women, 94% of people older than 60 years of age, and 95% of the immigrant population [18]. This trend reflects a broader movement towards public healthcare coverage among vulnerable and ageing populations.

The existing dual health system in Chile promotes health inequities for people with disabilities [19] since—due to their lower socioeconomic status—they primarily access the public health system, which is characterised by long waiting times, lack of specialised personnel, bureaucracy, and lack of personnel training and communication skills [20]. Evidence shows that persons with disabilities in Chile report lower utilisation of screening services, lower satisfaction with services, more barriers to accessing healthcare, and a higher likelihood of unmet needs compared to people without disabilities [21,22,23,24]. A recent health system analysis demonstrated gaps in the capacity of the health workforce to meet the needs of this population, highlighting a lack of nationally-implemented curricula and explicit training related to disability for healthcare professionals, as well as a lack of surveys or qualitative data on satisfaction disaggregated by disability [25]. 

The literature regarding patient satisfaction with health facility personnel is quite limited, and even more so concerning the satisfaction of older people with disabilities. Given the strong association between ageing and disability—reflected in the increasing prevalence of disability with age—alongside the demographic shift toward ageing populations and the greater health needs of older people with disabilities, it is essential to understand how this group experiences interactions with health facility personnel. Comparing younger and older people with disabilities allows us to identify potential age-related disparities in satisfaction with health facility personnel, which may reflect differences in expectations, communication needs, or healthcare experiences. Such comparisons can inform more tailored strategies to improve service responsiveness and equity across the life course for people with disabilities.

Evidence, however, on age-related differences is very limited and inconclusive: some studies have shown that older people report less positive experiences and satisfaction than younger patients, especially concerning measures of communication [26], while others have shown that age significantly moderates relations between trust in physicians and patient satisfaction, with positive relations becoming stronger with increasing age [27]. Few studies have shown that age is not a significant predictor of patient satisfaction [28]. To the best of our knowledge—and after a PubMed search using the general terms ‘disability’, ‘satisfaction’, and ‘healthcare staff’—this is the first study focusing on the satisfaction of people with disabilities with health facility personnel that includes non-healthcare staff, such as administrative personnel, in the Chilean and international context.

The aim of this study is to explore the satisfaction of persons with disabilities in Chile with health facility personnel. In this article, we use the term health facility personnel to refer both to healthcare workers (i.e., personnel providing healthcare services) and other types of workers who might interact with service users, including reception, maintenance, and security staff. Since one of Chile’s health objectives for the 2021–2030 decade is to reduce health inequities [29], it is important that the needs of persons with disabilities are taken into account and their perception of satisfaction with the system is explored in order to ensure that the system works for everybody.

The study has two objectives: (1) to analyse the socioeconomic and health-related differences between younger (18–59 years-of-age) and older (60+) people with disabilities, concerning their satisfaction with health facility personnel; and (2) to determine whether there is a significant difference concerning patient satisfaction between younger and older people with disabilities.

## 2. Materials and Methods

### 2.1. Data

This study employs data from the 2024 Disability and Citizenship (DISCA) Survey. This survey was conducted by the Millennium Nucleus Studies on Disability and Citizenship, a research centre funded by the National Agency for Research and Development (ANID) of the Ministry of Science, Technology, Knowledge, and Innovation of Chile. The objective of the DISCA survey was to explore the perceptions and experiences of adult people with disabilities living across Chile regarding political participation, healthcare access, and sexuality and reproduction. The survey was available online from August to November 2024. REDCap—a secure web application for building and managing online surveys and databases—was used. A total of 1074 individuals with disabilities participated in the study, substantially exceeding the minimum required sample size of 227. This larger sample was intentionally included to facilitate disaggregated analyses by type of disability, ensuring more detailed and reliable insights across different disability categories.

The general coverage of the DISCA survey was the adult population with disabilities living in private households and residing in the territory of the country. The sampling was non-probabilistic and by convenience. The survey was constructed with the participation of an Experts by Experience Committee, which included people with different kinds of disabilities, acting in a consulting role for the DISCA research centre. The survey was validated for its use in Chile (content validity index = 0.82). 

The DISCA survey was available in two versions: easy language and easy language with explanation of various terms; the latter version was approved by a non-governmental organisation of people with intellectual disability (Agrupación Líderes con Mil Capacidades). Respondents could access the survey either by phone or by computer. Before beginning, they were required to watch an informational video about the survey, which included captions and sign language interpretation. After viewing the video, participants proceeded to review and sign the informed consent form.

On average, the completion of the entire survey took approximately 25 min. While the vast majority of the surveys were conducted online with people with disabilities undertaking the survey themselves, approximately 5% of respondents declared that they needed assistance (before the start of the survey, people indicated whether they needed assistance). Such assistance was primarily provided by telephone and communication platforms by DISCA’s trained personnel; in certain cases, assistance was provided face-to-face. 

The DISCA survey included 66 variables in total and had 5 modules: (a) socioeconomic characterisation: 13 variables; (b) disability and support: 5 variables; (c) political participation: 14 variables; (d) access to healthcare: 23 variables; and (e) sexuality and reproduction: 11 variables. 

The authors confirm that all procedures and analyses in this work comply with the ethical standards of the relevant national committees regarding research on humans and with the Helsinki Declaration of 1975, as revised in 2008. The research received the ethical approval of the Ethics Research Committee of Diego Portales University, Chile (ID: 042-2023).

### 2.2. Variables

Our study adopts the definition of an older person, as employed by the National Service for the Elderly (SENAMA, from its initials in Spanish), based on Law 19828 (2002), which specifies that “…for all legal effects, older person is a person that has reached 60 years of age” [30]. In this study, ‘younger people’ are those aged 18 to 59, and ‘older people’ are those above 60 years of age. As we explore access to healthcare for older people, the variables used are derived from modules 1 (Socioeconomic characterisation), 2 (Disability and support), and 4 (Access to healthcare) of the DISCA survey. 

There are five dependent variables, measuring people with disabilities’ satisfaction with different health facility personnel. ‘Patient satisfaction’ is based on “…whether a patient’s expectations of what should happen were met” [31]. The question in the survey was: “If you had a consultation, control or medical attention in the last 12 months, how would you evaluate your satisfaction with the people that attended you?”. The health facility personnel included (a) doctors; (b) other health professionals (for example, physical therapists, nurses, etc.); (c) paramedical technicians; (d) administrative personnel (for instance, secretaries, etc.); and (e) security guards and cleaning personnel. For each of these categories, the possible answers were ‘excellent’, ‘good, ‘neither good nor bad’, ‘bad’, and ‘very bad’. The answers were grouped together into three: ‘high satisfaction’ (includes ‘excellent’ and ‘good’ evaluations), ‘average satisfaction’ (includes ‘neither good nor bad’ evaluations), and ‘low satisfaction’ (includes ‘bad’ and ‘very bad’ evaluations). 

Disability in the survey was self-reported. The categorisation of different types of disability was formulated together with the Experts by Experience Committee, and followed the definition of disability, according to the Article 1 of the CRPD, which specified that persons with disabilities “include those who have long-term physical, mental, intellectual or sensory impairments which in interaction with various barriers may hinder their full and effective participation in society on an equal basis with others” [32]. The question was “How do you identify yourself?”, with the following possible answers: person with physical disability, blind person or person with visual disability, deaf person or person with hearing disability, person with intellectual disability, person with developmental disability, person with psychosocial or mental health disability, blind and deaf person, and other type of disability. People could choose more than one possible answer. 

The socioeconomic variables included were (a) age: 18–59/60+; (b) gender: men/women; (c) geographical region: urban/rural; (d) education: primary/secondary/tertiary/special; and (e) indigeneity: indigenous/not indigenous. The health-related variables included the following: (a) health self-assessment: bad/neither good nor bad/good; (b) chronic disease: yes/no; and (c) health insurance: public (FONASA)/private (ISAPRE).

There were very few missing data points in the dataset. On the one hand, the number of observations of the dependent variables fluctuated, as not all people had contact with different hospital staff members; for example, the variable “satisfaction with paramedical personnel” had 635 observations, as the rest of the people mentioned that they had no interaction with this type of personnel. This ranged from 911 observations (satisfaction with doctors) to 618 observations (satisfaction with security guards and cleaning personnel). On the other hand, there were very few missing data for the socioeconomic and health-related variables: health insurance (3.7% missing), chronic disease (6.6% missing), indigeneity (6.0% missing), and geographical zone (0.09% missing). Case deletion (default in Stata), which analyses cases with available data on each variable, was minimal. Since we have a large enough sample, the statistical power is considered sufficiently high [33].

### 2.3. Statistical Analysis

Chi-square tests were applied in order to identify the socioeconomic and health-related differences between younger and older people with disabilities with regard to their satisfaction with health facility personnel (Objective 1). Ordered logistic regressions were employed to predict the ordinal dependent variables (that is, satisfaction with health facility personnel) given one (simple regression) or more independent variables (multiple regression) (Objective 2).

We adopted the Strengthening the Reporting of Observational Studies in Epidemiology (STROBE) guidelines [34] (Table S1, Supplementary Material). All statistical analyses were performed using the STATA©15 program.

## 3. Results

**Figure 1:**
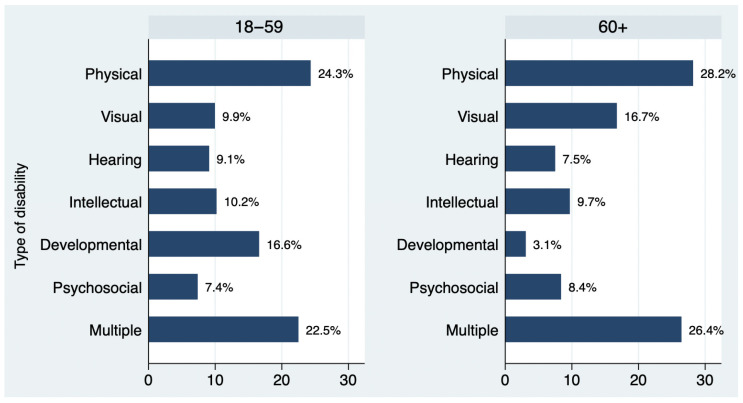
Younger (18–59 years of age) and older (60+) people with disabilities in the sample, according to type of disability.

Out of 1074 people with disabilities who participated in the survey, 832 (77.5%) participants were between 18 and 59 years of age, and 242 (22.5%) people were older than 60 years of age. Figure 1 presents the two age groups per type of disability. The differences are statistically significant.

Table 1 summarises the characteristics of younger (18–59 years of age) and older (60+) people with disabilities in our sample. Concerning socioeconomic variables, there are more younger women with disabilities in our sample (63.6%) than older women (50%). With regard to education, most younger people with disabilities have a tertiary education (59.6%), while 6.5% have special education; these percentages are 33.5% and 0.8%, respectively, for older people. Regarding health-related variables, there are more older people with disabilities who evaluate their health as bad (38.0%) than younger people (14.9%). There is also a higher percentage of older people with chronic diseases (90.8%) in comparison to younger people (60.0%). Additionally, more older people are affiliated with the public health insurance (94.9%) than younger people (84.5%). 

Concerning the experiences with healthcare facility staff, there is a marked difference between younger and older people with disabilities in the sample. For all variables, there is a higher percentage of older people that have declared high satisfaction with (a) doctors (93.9% of older people vs. 79.9% of younger people); (b) other health professionals (94.9% vs. 83.2%); (c) paramedical technicians (93.2% vs. 73.4%); (d) administrative personnel (85.8% vs. 63.7%); and (e) security guards and cleaning personnel (89.3% of older people vs. 78.5% of younger people). All these differences are statistically significant.

Simple and multivariable ordered logistic regressions were performed to examine whether there is a difference in the satisfaction of younger and older people with disabilities with regard to health facility personnel. The simple regression included only the variable ‘age’, while the multivariable regression included all the socioeconomic and health-related variables.

Table 2 presents the results of the ordered logistic regressions. The results show that for older people with disabilities, the odds of evaluating their satisfaction with health facility personnel as ‘high’ vs. the combined evaluation of ‘average’ and ‘low’ are from 3.1 to 4.7 times greater in comparison to younger people with disabilities, given that all the other variables in the model are held constant.

More particularly, for older people with disabilities, the odds of evaluating their satisfaction with doctors as ‘high’ vs. evaluating it as ‘average’ and ‘low’ were 3.8 times greater than that of younger people with disabilities, maintaining all other variables constant. Concerning their satisfaction with other health professionals, the odds were 4.7 times greater, while regarding their satisfaction with paramedical technicians, they were 4.3 times greater. For older people with disabilities, the odds of evaluating their satisfaction with administrative personnel as ‘high’ vs. evaluating it as ‘average’ and ‘low’ were 3.1 times greater than for younger people with disabilities, all other variables constant. The OR for the satisfaction with security guards and cleaning personnel was not statistically significant.

There was no collinearity affecting the results, with mean variance inflation factor (VIF) ranging from 1.58 to 1.70. Interaction terms were also used in earlier models. In order to identify the models with the best fit, the Akaike information criterion (AIC) and the Bayesian information criterion (BIC) were employed. The models that were selected and presented here are those with the lowest AIC and BIC values, indicating a better fit. 

**Figure 2:**
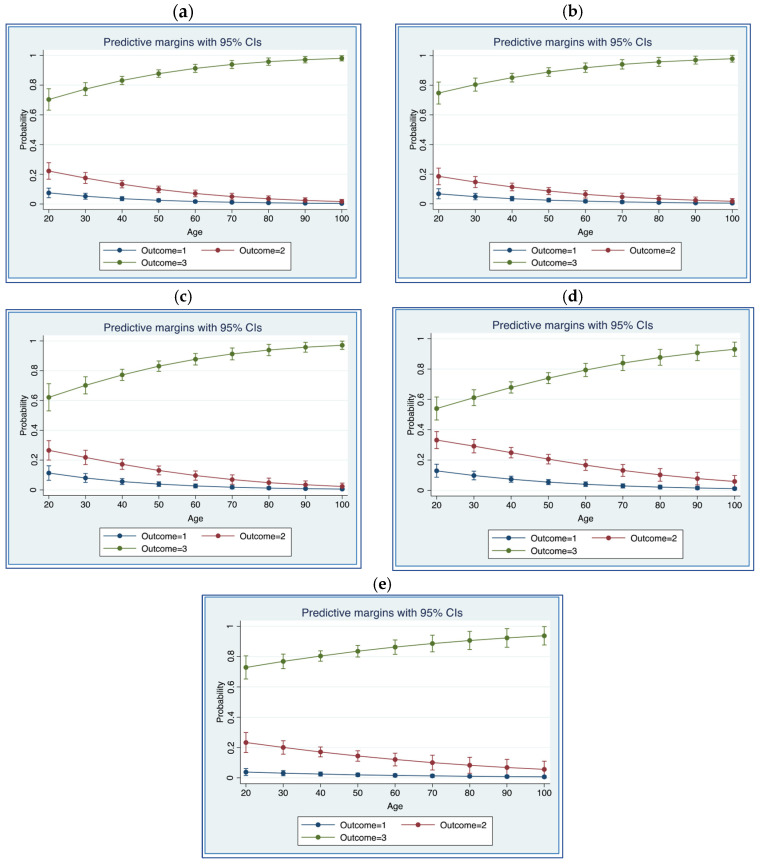
Predicted probabilities for ‘low satisfaction’ (Outcome 1), ‘average satisfaction’ (Outcome 2), and ‘high satisfaction’ (Outcome 3) with health facility personnel, when the predictor variable is ‘age’. (**a**) Predicted probabilities for ‘satisfaction’ with doctors; (**b**) predicted probabilities for ‘satisfaction’ with other healthcare personnel; (**c**) predicted probabilities for ‘satisfaction’ with paramedical technicians; (**d**) predicted probabilities for ‘satisfaction’ with administrative personnel; (**e**) predictive probabilities for ‘satisfaction’ with security guards and cleaning personnel.

Figure 2a–e present the predicted probabilities for satisfaction with health facility personnel when the predictor variable is ‘age’.

As it can be observed in all figures, the predicted probabilities for Outcome 3 (that is, “high satisfaction”) increase with age; the largest difference can be seen in Figure 2d (Satisfaction with administrative personnel), where the probability of being highly satisfied increases from 53.9% at the age of 20 to 80.3% at the age of 60 and 93.7% at the age of 90. A large difference can also be observed in Figure 2c (Satisfaction with paramedical technicians), where the probability of being highly satisfied increases from 62.1% at the age of 20 to 86.2% at the age of 60 and 93% at the age of 90. On the other hand, in all figures, the predicted probabilities for Outcome 2 (that is, “average satisfaction”) and Outcome 1 (that is, “low satisfaction”) decrease with age. 

## 4. Discussion

The findings of this study reveal significant differences in the satisfaction of younger and older people with disabilities regarding health facility personnel in Chile. Consistently, there was a higher percentage of older people with disabilities who reported high satisfaction with doctors, other health professionals, paramedical technicians, administrative personnel, and security guards and cleaning personnel compared to younger people with disabilities. These differences were statistically significant across all categories. Concerning the results of the ordered logistic regressions, these revealed that older people with disabilities have greater odds of evaluating their satisfaction with health facility personnel as ‘high’ in comparison to younger people with disabilities, with the exception being satisfaction with security guards and cleaning personnel, where the OR was not significant.

These results suggest that age plays a key role in shaping satisfaction with health facility personnel for people with disabilities, and this can also be observed in Figure 2a–e. In these figures, the predicted probabilities for evaluating satisfaction with health facility personnel as ‘high’ increase with age, on average, from approximately 67% at the age of 20 to 87% at the age of 60 and 96% at the age of 90.

Although evidence on this topic is mixed, our findings align with previous studies on patient satisfaction with health facility personnel that indicate that age is the most important and consistent factor of patient satisfaction among other sociodemographic variables [28,35]. These studies have revealed that older patients are generally more satisfied than younger patients; factors such as having a higher educational level, being native-born, and being in good or very good health condition are positively correlated with higher satisfaction for older patients [36]. Quality dimensions related to higher satisfaction of older patients include being treated well by doctors, being able to participate in decisions, having enough time with doctors, and being given clear medical information [28].

Our results agree with the limited evidence indicating a positive association between patient satisfaction with healthcare facility personnel and age [36,37,38]. The results also align with prior studies indicating that trust in physicians and satisfaction tend to increase with age, reinforcing the idea that older individuals may perceive healthcare encounters more positively [27]. This could be due to healthcare professionals using a more person-centred approach in their interaction with older patients [39]. The Person-Centred Care (PCC) model is a guiding principle of Chile’s Ministry of Health and the National Service for the Elderly (SENAMA) and is applied across programmes serving large numbers of older people, including those with disabilities and geriatric syndromes [40]. The PCC model emphasises physical and emotional well-being; respect for individual preferences; and the promotion of independence through supportive environments, protection of privacy, personal identity, and social inclusion, with a strong focus on dignity in older age. These principles contribute to enhancing the autonomy, functionality, and quality of life of older people. Professional teams may apply this person-centred approach—explicitly or implicitly—when working with older people with disabilities, which can in turn lead to higher levels of satisfaction with the health personnel who care for them.

Another possible explanation for these findings is that older people with disabilities may have lower expectations of healthcare services or different evaluation criteria compared to younger individuals, leading them to report higher satisfaction levels despite potential shortcomings in care [41]. Previous research has suggested that satisfaction is influenced not only by the quality of services but also by service users’ expectations, which may be shaped by their past experiences and perceived alternatives [41]. Indeed, experiences of adversity during their lifetime may have equipped older people with more ‘resilience’, i.e., more positive attitudes towards received healthcare [42].

Additionally, the increased interaction with the healthcare system due to chronic conditions in older individuals may foster stronger patient-provider relationships and a greater knowledge on how to navigate the healthcare system, potentially improving satisfaction levels [37]. In the particular case of Chile, the existence of Community Rehabilitation Centres, which use community-based rehabilitation strategies and provide services to people with disabilities, including a high percentage of older people with disabilities, may contribute to higher levels of satisfaction with the services provided, as these centres promote person-centred care, community participation, and continuity of support [43]. On the other hand, younger individuals with disabilities may have higher expectations regarding efficiency, accessibility, and communication within healthcare facilities, which could contribute to their lower satisfaction levels.

These findings highlight the need for targeted interventions to improve the healthcare experiences of people with disabilities, including staff training on disability-inclusive care and improvements in healthcare facility accessibility. A recent study revealed that out of 714 practicing US physicians nationwide, only 40.7% were very confident about their ability to provide the same quality of care to patients with disabilities, just 56.5% strongly agreed that they welcomed patients with disability into their practices, and 18.1% strongly agreed that the health care system often treats these patients unfairly; these results reveal persistent health disparities affecting people with disabilities, compounded by potentially biased views among physicians [44].

Policymakers and healthcare providers should focus on ensuring that all individuals, regardless of age, receive high-quality, equitable, and dignified care. Health workers should be provided with evidence-based training and professional development in order to improve their knowledge, confidence, self-efficacy, and competence to support patients with disabilities. Such training, community placements, simulations, and interactive sessions were found to be most effective when people with disabilities were involved [45]. Furthermore, given the growing recognition of the role of non-medical personnel in shaping healthcare experiences, targeted training programs for administrative, security, and cleaning staff could improve service user satisfaction across all age groups. Making health care more inclusive of people with disabilities will improve health for other groups (such as older people) and reduce health system costs; cost–benefit analyses have shown that the advancement of disability inclusion in the health sector is an investment with dividends [1].

One of the main aspects of disability-inclusive health systems is the existence of well-trained staff with the skills and knowledge to provide appropriate and quality care [46]. On the one hand, the Inter-American Convention on the Protection of the Human Rights of Older Persons (ratified by Chile) specifies respectful treatment and preferential care for older persons and states that the social and health services they receive must be of high quality [47]. On the other hand, Article 25d of the CRPD specifies that health professionals should offer the same quality of care to persons with disabilities as to others and receive appropriate training [48]. Nevertheless, studies have indicated that health workers need to improve their confidence, competency, attitudes, and comfort in treating patients with disabilities [44,45,49].

Inadequately or inappropriately trained healthcare workers can represent one of the biggest barriers affecting access to and satisfaction with healthcare services for persons with disabilities [1]. Healthcare workers’ general lack of disability training, lack of awareness of relevant policies and guidelines, and inability to adapt public health measures to cater to the needs of people with disabilities, especially during health emergencies, can lead to negative experiences, miscommunication, and low use of services by this population subgroup [50]. Despite calls to integrate and/or strengthen disability training into health worker curricula [51,52], such efforts remain insufficient and largely dependent on the priorities, resources, and commitments of individual countries and educational institutions, leading to significant variability in implementation and impact.

While disability training is key for achieving health equity for people with disabilities, it is equally important to address the shortage of health professionals, a shortage that will only worsen if we take into account the demographic transition occurring at a global level. The WHO estimates a shortage of at least 600,000 health professionals in Latin America and the Caribbean by 2030, based on the target of 44.5 professionals (medical, nursing, and midwifery personnel) per 10,000 population [53]. It is important, therefore, to address both the lack of a sufficient number of health professionals but also to provide them with adequate training—including in disability and emergency situations—in order to reduce unmet healthcare needs, improve satisfaction with health services, and improve health systems’ resilience in the face of crises.

Although user satisfaction surveys have been developed, validated, and implemented within the Chilean health system—primarily in hospitals and primary healthcare centres (CESFAM)—they have not adequately addressed accessibility for people with disabilities or older people. Consequently, adjustments are necessary to ensure that these populations can participate meaningfully in such evaluations. This includes incorporating questions about the quality of user treatment received from health facility staff, ranging from physicians to security personnel. Moreover, there is a pressing need to advance the design, validation, and routine application of specialised user satisfaction surveys tailored to centres, programmes, and services catering to older adults with disabilities—such as community rehabilitation centres, day centres for older adults with mild to moderate dependency, and residential care facilities—as well as private clinics and medical centres. Integrating these evaluation tools into the regular practice of comprehensive geriatric assessment used by health professionals would foster a continuous improvement cycle, enhancing the quality of care provided to this population group [54].

This study has several limitations that should be acknowledged. While Chile’s dual health system has been associated with disparities in access—particularly in the public sector due to long waiting times, limited availability of specialists, and bureaucratic hurdles—our analysis focused specifically on satisfaction with health facility personnel. We did not address other aspects, such as physical accessibility, financial constraints, or the availability of information and communication support, all of which shape the healthcare experiences of people with disabilities, particularly those who are older or from lower socioeconomic backgrounds. Another limitation of this study is that disability was self-reported and not based on the International Classification of Functioning, Disability, and Health (ICF). Also, as this study is observational, we cannot establish causality between patient satisfaction and other variables. Furthermore, since the sampling was not probabilistic, we cannot generalise the results for the entire adult population with disabilities in Chile. It was also not possible to include other variables or dimensions, for instance, reasons behind high or low satisfaction with health facility personnel, that could have shed more light on the results.

However, our research is important, as it is the first study that explores the satisfaction of younger and older people with disabilities with health facility personnel in Chile, a topic that has been generally very little studied in the Latin American region and internationally. To the best of our knowledge, it is also the only study that explores satisfaction with administrative staff as well as security guards and cleaning personnel. The few studies that exist—including those on general patient satisfaction—focus primarily on doctors and nurses. Anecdotal evidence from the experience of persons with disabilities associated with the DISCA research centre has revealed that they or other people with disabilities they know are more likely to experience problems or be less satisfied when dealing with non-healthcare personnel, such as administrative staff. It is thus essential to assess the satisfaction of individuals with disabilities regarding all types of personnel they may encounter when accessing and utilising healthcare services. Equally important is providing comprehensive disability awareness training to all staff—including non-clinical personnel such as administrative and security staff—to ensure respectful, inclusive, and accessible care throughout the healthcare experience.

Future research should further explore the underlying reasons for these age-related differences in satisfaction with health facility personnel, incorporating qualitative approaches to capture personal experiences and expectations. Additionally, studies should investigate how different dimensions of satisfaction—such as perceived respect, accessibility, and communication—vary by age, disability type, and category of health facility personnel. Addressing these gaps could contribute to the development of more inclusive healthcare systems that effectively respond to the diverse needs of people with disabilities across different life stages.

## 5. Conclusions

This study highlights the significant differences in satisfaction with health facility personnel between younger and older people with disabilities in Chile, reinforcing the role of age in shaping healthcare experiences. Older individuals with disabilities consistently reported higher satisfaction levels, which may be influenced by lower expectations, stronger patient–provider relationships, or greater familiarity with the healthcare system due to more frequent interactions. However, the lower satisfaction reported by younger individuals with disabilities underscores the need for targeted interventions that enhance communication, responsiveness, and the overall quality of interactions within healthcare settings. Given the critical role of non-medical personnel in shaping healthcare experiences, future policies should address gaps in training for administrative, security, and maintenance staff, in addition to healthcare professionals, to create a more inclusive and equitable health system.

While disability-inclusive training for health workers is essential for improving satisfaction with healthcare, structural challenges, such as workforce shortages and inequities in access, must also be addressed. Chile’s dual health system, characterised by long waiting times and bureaucratic hurdles in the public sector, exacerbates disparities faced by people with disabilities, especially older people and those with lower socioeconomic status. Strengthening disability training, integrating inclusive policies, and addressing systemic barriers will be key to reducing unmet healthcare needs and improving satisfaction among people with disabilities. Future research should explore the specific drivers of satisfaction across different disability types and age groups to inform evidence-based policies that promote equitable healthcare for all.

## Figures and Tables

**Table 1:** Sample characteristics.

Variable	People with Disabilities Aged 18–59 (n = 832, 77.5%)	People with Disabilities Aged 60+ (n = 242, 22.5%)	*p*
**Socioeconomic variables**			
Gender			
Men	303 (36.4%)	121 (50.0%)	*p* < 0.001
Women	529 (63.6%)	121 (50.0%)	
Age (median, IQR)	36 (28–45)	68 (64–73)	
Zone			
Urban	685 (82.4%)	202 (83.5%)	*p* = 0.707
Rural	146 (17.6%)	40 (16.5%)	
Education			
Primary	60 (7.2%)	53 (21.9%)	*p* < 0.001
Secondary	222 (26.7%)	106 (43.8%)	
Tertiary	496 (59.6%)	81 (33.5%)	
Special	54 (6.5%)	2 (0.8%)	
Indigeneity			
Not indigenous	694 (89.1%)	216 (93.9%)	*p* = 0.031
Indigenous	85 (10.9%)	14 (6.1%)	
**Health-related variables**			
Health self-assessment			
Bad	124 (14.9%)	92 (38.0%)	*p* < 0.001
Neither good nor bad	335 (40.3%)	97 (40.1%)	
Good	373 (44.8%)	53 (21.9%)	
Chronic disease			
No	305 (40.0%)	22 (9.2%)	*p* < 0.001
Yes	458 (60.0%)	218 (90.8%)	
Health insurance			
Public (FONASA)	676 (84.5%)	222 (94.9%)	*p* < 0.001
Private (ISAPRE)	124 (15.5%)	12 (5.1%)	
**Satisfaction with health facility personnel**			
With doctors			
Low	36 (5.3%)	2 (0.9%)	*p* < 0.001
Average	101 (14.8%)	12 (5.3%)	
High	546 (79.9%)	214 (93.9%)	
With other health professionals			
Low	23 (4.1%)	2 (1.3%)	*p* = 0.001
Average	71 (12.7%)	6 (3.8%)	
High	467 (83.2%)	149 (94.9%)	
With paramedical technicians			
Low	30 (6.3%)	2 (1.2%)	*p* < 0.001
Average	96 (20.3%)	9 (5.6%)	
High	347 (73.4%)	151 (93.2%)	
With administrative personnel			
Low	58 (9.8%)	3 (1.8%)	*p* < 0.001
Average	156 (26.4%)	21 (12.4%)	
High	376 (63.7%)	145 (85.8%)	
With security guards and cleaning personnel			
Low	16 (3.1%)	1 (1.0%)	*p* = 0.038
Average	95 (18.5%)	10 (9.7%)	
High	404 (78.5%)	92 (89.3%)	

**Table 2:** Ordered logistic regressions on ‘high’ satisfaction with health facility personnel by older people with disabilities.

Dependent Variable	OR	95% CI	Observations	LR chi^2^ (*p*)
**Satisfaction with doctors**				
Simple regression	3.864	2.181–6.845	911	28.76 (*p* < 0.001)
Multiple regression	3.831	1.950–7.524	790	78.27 (*p* < 0.001)
**Satisfaction with other health professionals**				
Simple regression	3.741	1.777–7.880	718	16.49 (*p* < 0.001)
Multiple regression	4.655	1.878–11.541	612	55.36 (*p* < 0.001)
**Satisfaction with paramedical technicians**				
Simple regression	4.992	2.619–9.512	635	33.71 (*p* < 0.001)
Multiple regression	4.305	2.074–8.936	549	69.98 (*p* < 0.001)
**Satisfaction with administrative personnel**				
Simple regression	3.504	2.208–5.561	759	34.66 (*p* < 0.001)
Multiple regression	3.126	1.824–5.357	651	90.06 (*p* < 0.001)
**Satisfaction with security guards and cleaning personnel**				
Simple regression	2.309	1.195–4.463	618	7.33 (*p* = 0.007)
Multiple regression	1.776	0.839–3.758	516	24.79 (*p* = 0.010)

Note: Multiple regressions are adjusted for gender, geographical zone, education, indigeneity, health self-assessment, chronic disease, and health insurance.

## Data Availability

The dataset presented in this article is not readily available because the data are part of an ongoing study and because it contains information that could compromise the privacy of research participants. Requests to access the datasets should be directed to ESR.

## Supplementary Materials

The following supporting information can be downloaded at: https://www.mdpi.com/article/10.3390/ijerph22071103/s1, Table S1: Strengthening the Reporting of Observational Studies in Epidemiology (STROBE).

## Abbreviations

The following abbreviations are used in this manuscript:

AIC	Akaike information criterion
BIC	Bayesian information criterion
DISCA	Disability and citizenship
FONASA	National health fund
ISAPRE	Health insurance institutions
CRPD	Convention on the Rights of Persons with Disabilities
WHO	World Health Organization
